# Image Encryption via DDPM Skipping-Step Diffusion Based on a Six-Dimensional Hyperchaotic System with Feedback Control

**DOI:** 10.3390/e28070831

**Published:** 2026-07-22

**Authors:** Songran Wang, Hanqing Zhao, Wei He, Tao Wang, Zhiben Zhuang, Tianfu Zhang, Jiacheng Xu

**Affiliations:** 1 School of Mathematics and Computational Sciences, Huaihua University, Huaihua 418000, China; 2 College of Intelligent Systems Science and Engineering, Hubei Minzu University, Enshi 445000, China

**Keywords:** digital image encryption, hyperchaotic system, denoising diffusion probabilistic model (DDPM), bit-level scrambling

## Abstract

In this paper, we propose a novel image encryption algorithm that integrates a six-dimensional hyperchaotic system with the forward diffusion process of denoising diffusion probabilistic models (DDPMs). The proposed framework synergistically combines the hyperchaotic system’s high sensitivity to initial conditions with the DDPM’s Markov chain-based skipping-step diffusion mechanism, thereby enabling dual-level confusion-diffusion operations at both the pixel and bit levels. This dual-strategy approach significantly enhances plaintext sensitivity and ciphertext randomness. Comprehensive simulations demonstrate that the algorithm achieves superior performance in key space expansion, histogram uniformity, adjacent pixel correlation reduction, information entropy optimization, and resistance to differential attacks. The algorithm exhibits strong resilience against various attack vectors, including brute-force attacks, statistical analysis, chosen-plaintext attacks and common image degradation factors (e.g., noise contamination and cropping). These characteristics establish the proposed method as a highly secure and practical solution for image data protection in cloud-IoT environments.

## 1. Introduction

The proliferation of digital imaging technologies in cloud computing and IoT applications has exposed massive image data to escalating security threats during transmission and storage. Conventional cryptographic algorithms, such as AES and DES, are effective for textual data but demonstrate inherent limitations in processing digital images characterized by high data redundancy, strong pixel correlation, and low entropy [1,2]. Matrix transformation-based approaches such as the Arnold transform [3] and Baker mapping [4] use elementary geometric transformations to disrupt pixel arrangements. However, these methods suffer from periodicity issues that allow image recovery through repeated transformations, and their invariant histogram properties make them vulnerable to statistical analysis.

The application of chaotic systems in cryptography, pioneered by Matthews [5], has led to extensive research on chaos-based encryption schemes [6,7,8,9]. Traditional chaotic encryption uses sensitivity to the initial conditions and pseudo-randomness of low-dimensional chaotic systems (e.g., Logistic map and Lorenz system) for pixel permutation. However, these approaches encounter critical challenges, including period degradation in low-dimensional chaos and vulnerability to chosen-plaintext attacks caused by linear confusion structures.

Recent advancements in artificial intelligence have introduced novel paradigms for image encryption. Researchers have explored hybrid architectures that integrate chaotic systems with deep learning models [10]. Representative works include chaotic encryption with simple perceptrons [11], convolutional neural network (CNN)-integrated chaotic encryption [12], and multidimensional chaotic-CNN frameworks [13,14]. Despite this progress, a significant research gap remains in exploiting emerging generative models such as the denoising diffusion probabilistic model (DDPM) for cryptographic applications.

DDPMs represent a groundbreaking class of generative models that progressively transform data into Gaussian noise through a Markov chain-based forward diffusion process [15]. The reverse denoising process enables high-quality data generation, whereas the forward diffusion process possesses unique cryptographic properties: (1) path irreversibility: the diffusion process is theoretically irreversible without knowledge of the noise trajectory and (2) structured randomness: the Markov chain generates noise sequences with high ergodicity and complex correlations.

In this paper, we present a novel encryption framework that synergistically integrates the DDPM forward process with a six-dimensional hyperchaotic system. We summarize the key contributions of this study as follows:

(1) Markov Chain Skipping-Step Diffusion Mechanism: We innovatively adapt the DDPM forward diffusion process for encryption by introducing a skipping-step strategy. Unlike the standard DDPM, which requires extensive iterations for generation, the proposed method selectively executes critical diffusion steps guided by hyperchaotic sequences. This strategy enables rapid and controllable mapping from plaintext to the noise domain while reducing computational overhead. Importantly, the skipping intervals and paths are determined by the hyperchaotic system, which introduces additional key dimensions and enhances security.

(2) Hyperchaos-Driven Anisotropic Diffusion: The Gaussian noise injection in the skipping-step process is dynamically modulated by sequences generated by the six-dimensional hyperchaotic system. This transforms the diffusion process from isotropic to anisotropic, thereby introducing spatial and channel-specific variations in noise characteristics. The resulting complexity in plaintext-ciphertext relationships significantly strengthens resistance to differential and statistical attacks.

(3) Dual-Domain Confusion-Diffusion Architecture: A hierarchical encryption structure is established through synergistic operations at both the pixel and bit levels. In the pixel domain, hyperchaos-driven diffusion ensures the global dispersion of information. At the bit level, bit-plane scrambling is applied using hyperchaotic sequences to achieve the precise rearrangement of individual bits. This dual-level approach ensures the comprehensive concealment of statistical and structural features of the plaintext.

(4) Security-Efficiency Synergy: By leveraging the skipping-step mechanism and high-dimensional chaos, the algorithm achieves a balance between security and computational efficiency. The reduced diffusion steps make it suitable for real-time applications, and the hyperchaotic system provides a robust source of randomness with an expansive key space, thereby ensuring resilience against brute-force attacks and chaos-based cryptanalysis.

To summarize, this study represents a significant advancement in image encryption by reinterpreting the theoretical foundations of DDPM for cryptographic purposes. Through the integration of hyperchaotic systems, bit-level operations, and the skipping-step diffusion mechanism, the proposed framework offers a novel, secure, and practical solution for protecting digital images in cloud-IoT environments.

The remainder of this paper is organized as follows: In Section 2, we introduce the foundational concepts. In Section 3, we describe the six-dimensional hyperchaotic system. In Section 4, we explain the proposed encryption algorithm. In Section 5, we present the simulation results and performance analysis. Finally, in Section 6, we report the conclusions.

## 2. Preliminaries

### 2.1. SHA-256

SHA-256 is a one-way Hash function, which has a pivotal position in the field of cryptography and information security, and its main role is to convert a message of arbitrary length into a message digest of 256-bit length. Therefore, it can map different images to different information digests and enhance the randomness of image encryption. The SHA-256 algorithm has four features: (1) the input length is variable, but the output length is fixed; (2) operation efficiency is high and the hash value can be calculated quickly; (3) different messages must have different message digests; and (4) the message digests cannot be inverted to the message [16].

### 2.2. The Mathematics of DDPM Forward Diffusion

The amount of noise added at each step of the forward diffusion process is very small; hence, it can be assumed that the difference between the previous image and the subsequent image is very small. This small change can be modeled well by a Gaussian distribution; hence, $q(x_{t}|x_{t-1})$ is used to indicate that a small amount of noise has been added, and the added noise $\varepsilon_{t}∼N\left(0,I\right)$ belongs to Gaussian noise.

The slightly noisier image $x_{t}$ in the forward diffusion process is computed based on the noisier image $x_{t-1}$ in the previous step; that is, the final pure noise $x_{T}$ is sampled from the conditional distribution $q(x_{t}|x_{t-1})$. Thus, the process of adding Gaussian noise gradually to the original image $x_{0}$ can be represented as

(1) $$x_{1}∼q\left(x_{1}|x_{0}\right),\;x_{2}∼q\left(x_{2}|x_{1}\right),\;\ldots ,\;x_{T}∼q\left(x_{T}|x_{T-1}\right).$$

**Figure 1:**
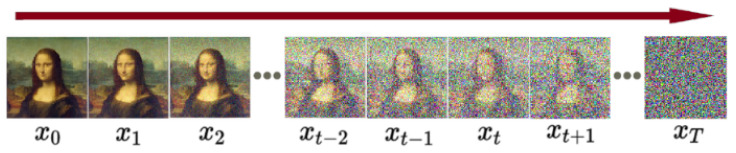
Schematic diagram of the forward diffusion process.

At the end of the noise addition, $x_{t}$ becomes pure Gaussian noise with no information about the original image. Figure 1 illustrates this process.

The variance scheduling table β is a set of predefined $\beta_{t}$ values that determines how much noise is added at each step of the diffusion process, where $\beta_{t}$ has to satisfy $0<\beta_{1}<\beta_{2}<\ldots <\beta_{T}<1$. $\beta_{t}$ is not constant at each time step *t*. For example, it can be linear, cosine, sigmoid, or quadratic scheduling.

In the original DDPM paper, the authors proposed linear scheduling, whereas Nichol et al. introduced the use of $\beta_{t}$ values in a cosine scheduling form. The core idea of cosine scheduling is that $\beta_{t}$ values vary over time steps according to a cosine function. This approach offers the advantage of smoothing the noise addition process at both ends [17]. Based on the above, to enhance the randomness of the encryption process, we input the hyperchaotic sequence generated by the hyperchaotic system into the improved cosine scheduling formula to control the value of the scheduling parameter β sequence.

The process of forward diffusion gradually adds a certain amount of Gaussian noise to the original image $x_{0}$ for t=1,…,T time steps, thereby generating a probability distribution of $x_{t-1}$ as follows:

(2) $$q\left(x_{t}|x_{t-1}\right)=N\left(x_{t};\sqrt{1-\beta_{t}}x_{t-1},\beta_{t}I\right).$$

The proportion of diffusion is controlled by the variance $\beta_{t}$ at each time step *t*. The variable $x_{t}$ depends only on the Gaussian noise $\varepsilon_{t}$ added by $x_{t-1}\cdot q\left(x_{t}|x_{t-1}\right)$ at each time step. The diffusion equation is

(3) $$x_{t}=\sqrt{1-\beta_{t}}x_{t-1}+\sqrt{\beta_{t}}\varepsilon_{t-1}.$$

In practical calculations, the reparameterization technique is typically used. Instead of computing $x_{1},x_{2},\ldots ,x_{t}$ step by step, we can directly compute $x_{t}$ after adding noise at any moment using only the known variance scheduler β and Gaussian noise $\varepsilon_{t}∼N\left(0,I\right)$. Let $\alpha_{t}=1-\beta_{t}$ and ${\bar{\alpha }}_{T}=\prod_{i=1}^{n}\alpha_{i}$.

Bringing $\alpha_{t}$ into the diffusion Equation (3) yields the following equation:

(4) $$x_{t}=\sqrt{\alpha_{t}}x_{t-1}+\sqrt{1-\alpha_{t}}\varepsilon_{t-1}.$$

Then, we obtain

(5) $$x_{t-1}=\sqrt{\alpha_{t-1}}x_{t-2}+\sqrt{1-\alpha_{t-1}}\varepsilon_{t-2}.$$

By the Equations (4) and (5),

(6) $$x_{t}=\sqrt{\alpha_{t}\alpha_{t-1}}x_{t-2}+\sqrt{\alpha_{t}\left(1-\alpha_{t-1}\right)}\varepsilon_{t-2}+\sqrt{1-\alpha_{t}}\varepsilon_{t-1}.$$

As we know, two independent Gaussian distributions are added together and the result is still a Gaussian distribution; that is, if $X∼N\left(\mu_{X},\sigma_{X}^{2}\right)\;,Y∼N\left(\mu_{Y},\sigma_{Y}^{2}\right)$, then $X+Y∼N(\mu_{X}+\mu_{Y},\sigma_{X}^{2}+\sigma_{Y}^{2})$, where *X* and *Y* are random variables.

If we combine $\sqrt{\alpha_{t}\left(1-\alpha_{t-1}\right)}\times \varepsilon_{t-2}$ and $\sqrt{1-\alpha_{t}}\times \varepsilon_{t-1}$ into a new Gaussian distribution, its variance is

(7) $$\alpha_{t}\left(1-\alpha_{t-1}\right)+\left(1-\alpha_{t}\right)=1-\alpha_{t}\alpha_{t-1}.$$

Therefore, its standard deviation is $\sqrt{1-\alpha_{t}\alpha_{t-1}}$ and the Gaussian term obtained after merging is $\sqrt{1-\alpha_{t}\alpha_{t-1}}\varepsilon^{\prime }$; hence, $x_{t}$ can be rewritten as

(8) $$x_{t}=\sqrt{\alpha_{t}\alpha_{t-1}}x_{t-2}+\sqrt{1-\alpha_{t}\alpha_{t-1}}\varepsilon^{\prime },\varepsilon^{\prime }∼N\left(0,I\right).$$

This is the continuation of the calculation that expands this chain, which can be expanded all the way up to $x_{0}$, expanding as follows:

(9) $$x_{t}=\sqrt{\alpha_{t}\alpha_{t-1}\ldots \alpha_{1}}x_{0}+\sqrt{1-\alpha_{t}\alpha_{t-1}\ldots \alpha_{1}}\varepsilon_{0}^{\prime }.$$

Finally, the following is obtained after the application of the reparameterization technique:

(10) $$x_{t}=\sqrt{{\bar{\alpha }}_{T}}x_{0}+\sqrt{1-{\bar{\alpha }}_{T}}\varepsilon .$$

Therefore, instead of gradual diffusion from $x_{0},x_{1},x_{2},\ldots ,x_{t}$, we generate $x_{0}$ directly from $x_{0}$ for any time step. Unlike the reparameterization strategy of the standard DDPM, our proposed strategy has a β sequence construction mechanism based on a continuous product to reduce the time of the encryption and decryption processes. To increase the randomness of encrypted images, we require that the jump rules of Markov chains must be satisfied during this process.

### 2.3. Box–Muller Algorithm

The Box–Muller Algorithm can efficiently generate random numbers with mean 0 and variance 1. It entails mapping two independent uniformly distributed random variables into two independent normally distributed random variables by varying the polar coordinates. The main process is to first obtain two independent sequences $U_{1}$ and $U_{2}$, and then compute theirs intermediate variables *R* and θ using the formula

(11a) $$\begin{array}{c} R=\sqrt{-2\ln U_{1}}; \end{array}$$

(11b) $$\begin{array}{c} \theta =2\pi U_{2}. \end{array}$$

In this case, the intermediate variable *R* obeys a Rayleigh distribution and the intermediate variable θ obeys a uniform distribution on [0,2π).

The trigonometric functions are then used to convert $U_{1}$ and $U_{2}$ to normal distributions in the following manner:

(12a) $$\begin{array}{c} Z_{0}=R\cos\theta ; \end{array}$$

(12b) $$\begin{array}{c} Z_{1}=R\sin\theta . \end{array}$$

The final generated $Z_{0}$ and $Z_{1}$ are independent and obey N(0,1).

### 2.4. Bit-Plane Scrambling Method

Bit-plane scrambling is a method for disambiguating an image by splitting it into multiple bit planes according to the bits. It is based on the fact that a single pixel value of 0∼255 occupies 8 bits and an RGB image has three channels; hence, a color image can be split into 24-bit planes. The advantage is that not only can the disambiguation operation be performed in a single bit plane but the order of the 24-bit planes can also be exchanged to further uncorrelate the neighboring pixels and the three channels.

## 3. New Six-Dimensional Hyperchaotic System

Reference [18] introduces two feedback controllers based on the Lorenz system and constructs a new five-dimensional chaotic system. Its equations are as follows:

(13) $$\left\{\begin{array}{ccc} \dot{x} & = & ay-bx-c\sin(u),\\ \dot{y} & = & dx-exv^{2},\\ \dot{z} & = & fxy-gz,\\ \dot{u} & = & hyz-iu,\\ \dot{v} & = & ju-kv, \end{array}\right.$$

where a,b,c,d,e,f,g,h,i,j and *k* are control parameters. The maximum Lyapunov exponent of most of the reported hyperchaotic systems is less than 4, and the larger the maximum Lyapunov exponent, the better the randomness. Therefore, based on system (13), we add one controller *v* and construct a new six-dimensional hyperchaotic system as follows:

(14) $$\left\{\begin{array}{ccc} \dot{x} & = & ay-bx-c\sin(u),\\ \dot{y} & = & dx-exv^{2},\\ \dot{z} & = & fxy-gz,\\ \dot{u} & = & hyz-iu,\\ \dot{v} & = & ju-kv,\\ \dot{w} & = & ly, \end{array}\right.$$

where a,b,c,d,e,f,g,h,i,j,k and *l* are control parameters. Compared with system (13), system (14) has a more complex topological structure. Additionally, system (13) is only an ordinary chaotic system, whereas system (14) is a hyperchaotic system. Simultaneously, the maximum Lyapunov exponent of system (14) is greater than 5.7. The initial values used in Section 3.1, Section 3.2, Section 3.3 and Section 3.4 are $y_{0}=\left[0.1,0.2,0.1,0.1,0.3,0.2\right]$.

### 3.1. Lyapunov Exponential and Randomness Analysis

**Figure 2:**
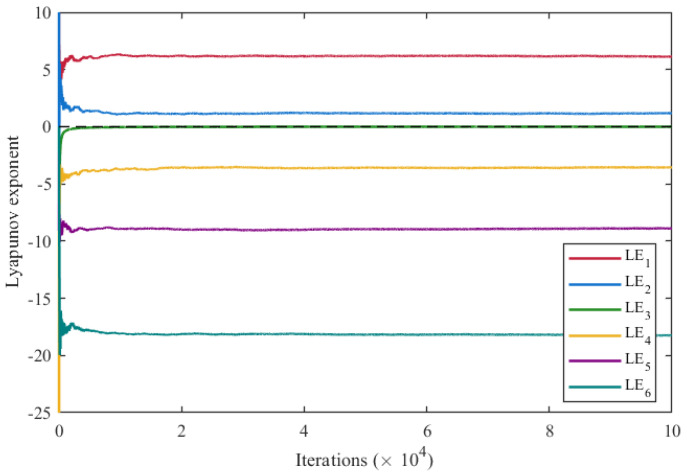
Lyapunov exponent diagram.

The primary dynamical properties of a chaotic system can be characterized by its Lyapunov exponent spectrum. For the parameter values a=10,b=11,c=1,d=31,e=1,f=1,g=11/3,h=1,i=3,j=1,k=9,l=1, the corresponding Lyapunov exponent spectrum is shown in Figure 2. Figure 2 demonstrates that the system is hyperchaotic, with two positive Lyapunov exponents of approximately 6 and 1, respectively, which indicates that the system exhibits strong randomness and high complexity.

The Lyapunov exponents are $\lambda_{1}=6.121584$, $\lambda_{2}=1.183124$, $\lambda_{3}=0$, $\lambda_{4}=-3.556723$, $\lambda_{5}=-8.894189$, and $\lambda_{6}=-18.248423$. The cumulative sums of the Lyapunov exponents are as follows:

$$\begin{array}{ccc} S_{1} & = & \lambda_{1}=6.121584,\\ S_{2} & = & \lambda_{1}+\lambda_{2}=7.304708,\\ S_{3} & = & \lambda_{1}+\lambda_{2}+\lambda_{3}=7.304708,\\ S_{4} & = & \lambda_{1}+\lambda_{2}+\lambda_{3}+\lambda_{4}=3.747985,\\ S_{5} & = & \lambda_{1}+\lambda_{2}+\lambda_{3}+\lambda_{4}+\lambda_{5}=-5.146204. \end{array}$$

Because $S_{5}$ is less than zero, we set j=4 and apply the Kaplan–Yorke formula $D_{KY}=j+\sum_{i=1}^{j}\lambda_{j}/|\lambda_{j+1|}$, which yields $D_{KY}=4.42095$. $D_{KY}$ is not an integer, which further illustrates the existence of singular attractors in the system (14) from a dimensional perspective, thereby indicating that the system is in a chaotic state. The dimension of the attractor is between 4 and 5, thereby indicating that the geometric complexity of the attractor is higher than four dimensions. We also conducted 0−1 tests on the hyperchaotic sequence. The results are shown in Table 1. The table shows that the hybrid system has good randomness.

### 3.2. Phase Diagram Analysis

**Figure 3:**
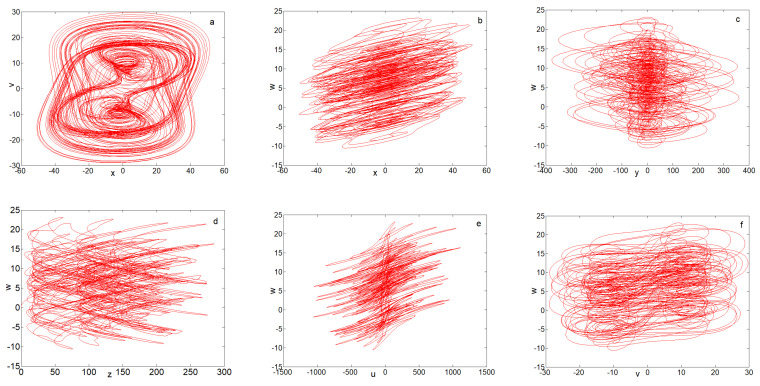
Two−dimensional plane phase diagram: (**a**) x−v flat; (**b**) x−w flat; (**c**) y−w flat; (**d**) z−w flat; (**e**) u−w flat; and (**f**) v−w flat.

**Figure 4:**
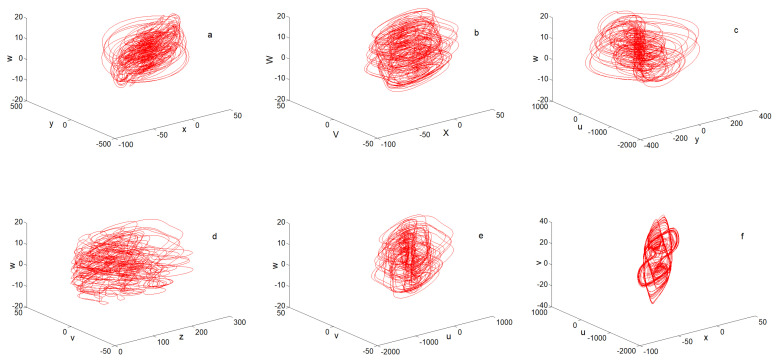
Three−dimensional phase diagram: (**a**) x−y−w space phase diagram; (**b**) x−v−w space phase diagram; (**c**) y−u−w space phase diagram; (**d**) z−v−w space phase diagram; (**e**) u−v−w space phase diagram; and (**f**) x−u−v space phase diagram.

For the parameter values a=10,b=11,c=1,d=31,e=1,f=1,g=11/3,h=1,i=3,j=1,k=9,l=1, the multi-band multi-wing two-dimensional (2D) and three-dimensional (3D) phase portraits generated by system (14) are presented in Figure 3 and Figure 4, respectively. These phase portraits demonstrate that the chaotic system generates multiple frequency bands and wings in various directions.

### 3.3. Time Series Chart

**Figure 5:**
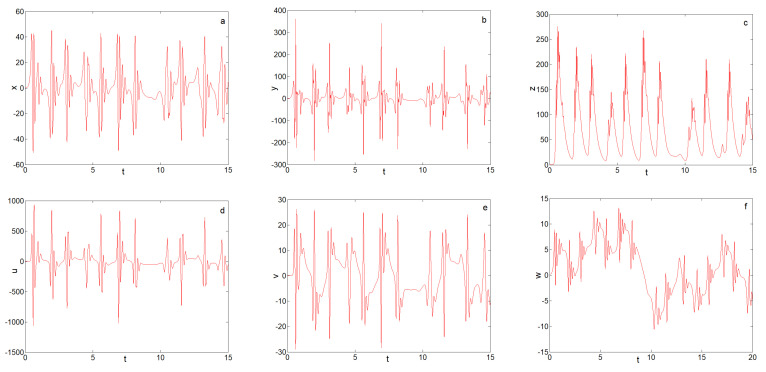
Time series diagram: (**a**) x−t time series; (**b**) y−t time series; (**c**) z−t time series; (**d**) u−t time series; (**e**) v−t time series; and (**f**) w−t time series.

For the parameter values a=10,b=11,c=1,d=31,e=1,f=1,g=11/3,h=1,i=3,j=1,k=9,l=1, the time series of x,y,z,u,v,w for system (14) are shown in Figure 5, which indicates that the system is in a chaotic state.

### 3.4. Poincaré Section

**Figure 6:**
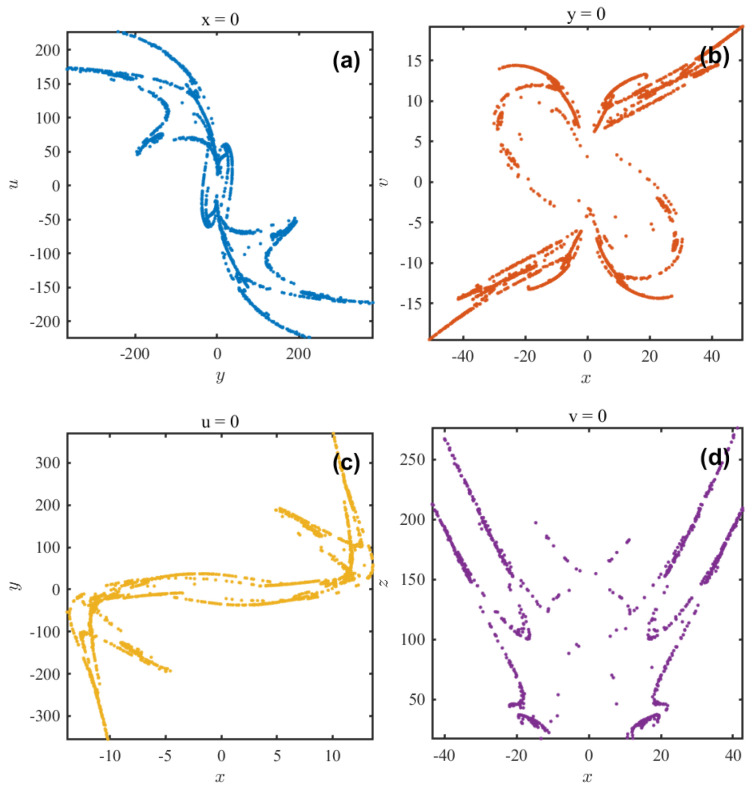
Poincaré interface diagram: (**a**) point passing through the y−u section in the positive direction when x=0; (**b**) point passing through the x−v section in the positive direction when y=0; (**c**) point passing through the x−y section in the positive direction when u=0; and (**d**) point passing through the x−z section in the positive direction when v=0.

A Poincaré section is formed by selecting a plane in the phase space and collecting the intersection points where the system trajectory crosses this plane in a prescribed direction. For the parameter values a=10,b=11,c=1,d=31,e=1,f=1,g=11/3,h=1,i=3,j=1,k=9,l=1, the Poincaré section of system (14) is taken at x=0,y=0,u=0,v=0, which retains only the crossings in the positive direction. The resulting section is presented in Figure 6.

## 4. Algorithm Descriptions

### 4.1. Encryption Process

**Figure 7:**
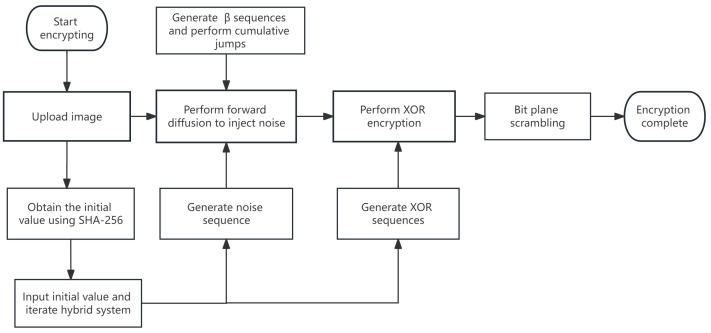
Flowchart of image encryption algorithm.

The flow chart of the encryption process is shown in Figure 7. Given an M×N×3 color plaintext image I, the encryption steps are as follows:

Step 1: Input the M×N×3 color plaintext image I and the customized string keyStr, and convert its keyStr to UTF-8 byte format.

Step 2: Read the binary data imgData of I and splice the keyStr to the front of imgData to obtain the spliced data cData.

Step 3: To ensure data integrity and prevent arbitrary tampering, use the SHA-256 algorithm to generate a 32 byte digest hash for cData.

Step 4: Extract the byte segments whose indices are located in the range of (i−1)×4+1 to i×4(1≤i≤8) in the hash and convert them to the 32-bit unsigned integer decV.

Step 5: Map decV to the range 0–10 and retain four decimals places; hence, the key sequence keys are generated. Select six of the eight values as the keys. The formula is

(15) $$key_{i}=\frac{\mathrm{dec}V}{\left(2^{32}-1\right)}\times 10.$$

Step 6: Output key sequence $keys=[key_{1},key_{2},key_{3},key_{4},key_{5},key_{6}]$. The key sequence keys are used as the initial condition of the chaotic system (14). Call the ODE45 program under the conditions of a relative tolerance of $10^{-3}$ and an absolute tolerance of $10^{-6}$. Set the step size to 0.01, discard the first 500 values, and calculate the total time $T_{1}=0.01\times \left(M\times N\times 3\right)+500$.

Step 7: Diffusion encryption is based on the probabilistic diffusion model for the forward diffusion process of noise injection. Set the total number of diffusion steps to T=800.

Step 8: Generate sequence β, where sequence β controls the noise increment at each time step. Use a chaotic system to generate T elements of sequence β, arranged in ascending order and satisfying the condition $0<\beta_{1}<\beta_{2}<\beta_{3}<\ldots <\beta_{T-1}<\beta_{T}<1$.

Step 9: Obtain the α sequence from α=1−β. The α sequence represents the mixing ratio of the data. Because the diffusion probability model is a Markov chain process, it can be used without iterative diffusion, and the closed-form solution at any time step can be obtained using the reparameterization technique to directly inject the final noisy image into images, whereupon we can pre-accumulate the concatenated multiplications and obtain the value ${\bar{\alpha }}_{T}$ of the cumulative T-steps of the α-sequence ${\bar{\alpha }}_{T}$ using ${\bar{\alpha }}_{T}=\prod_{i=1}^{T}\alpha_{i}$.

Step 10: Generate the noisy image. Use the key sequence keys as the initial value of the chaotic system (14) and select its first state variable *x* to generate the sequence to obtain the pseudo-random sequence $U_{1}$. Then, add a small perturbation to the initial value keys to ensure the independence of the pseudo-random sequence, obtain the initial value $k=[key_{1}+0.00001,key_{2},key_{3},key_{4},key_{5},key_{6}+0.00001]$, and generate the pseudo-random sequence $U_{2}$ with *k* as the initial value of the chaotic system (14), where $U_{2}$ is a hyperchaotic sequence generated by the sixth state variable w.

Step 11: Using sequences $U_{1}$ and $U_{2}$, generate the Gaussian-distributed noise sequence via the Box–Muller transform formula, and use the sequence N normalized to the range [0,1] to generate the noise image ε that accumulates up to T time steps. The noise image ε is also a color image of M×N×3. Repeat the above steps to generate sufficient noisy images.

Step 12: Inject noise. This process is a noise diffusion operation performed in the fractional domain. First, normalize the plaintext image I to the range [0,1] to obtain the value $X_{0}$ at step 0 of the diffusion process, and then diffuse the noise image ε accumulated up to the time step T into $X_{0}$. Thus, a closed-form solution of the time step T is obtained using the reparametrization technique; that is, the image $X_{T}$ after the injection of noise, which is defined in the process

(16) $$X_{T}=\sqrt{{\bar{\alpha }}_{T}}\times X_{0}+\sqrt{1-{\bar{\alpha }}_{T}}\times \varepsilon .$$

Step 13: In the confusion encryption stage, because confusion encryption is a differential encryption operation based on bitwise operations, first convert the image $X_{T}$ after noise injection to Uint8 format to obtain the adjusted image $I_{diff}$.

Step 14: Generate a sequence using key sequence keys as the initial values of the chaotic system (14) and select its second state variable y, mapping it to the [0,255] range and converting to Uint8 format to obtain the M×N×3 dissimilarity sequence S.

Step 15: Perform the XOR operation on the pixel values of all three channels of the image using the XOR operation:

(17) $$I_{xor}=I_{diff}⊕S.$$

Step 16: Get the final image $I_{xor}$ converted to Unit8.

**Figure 8:**
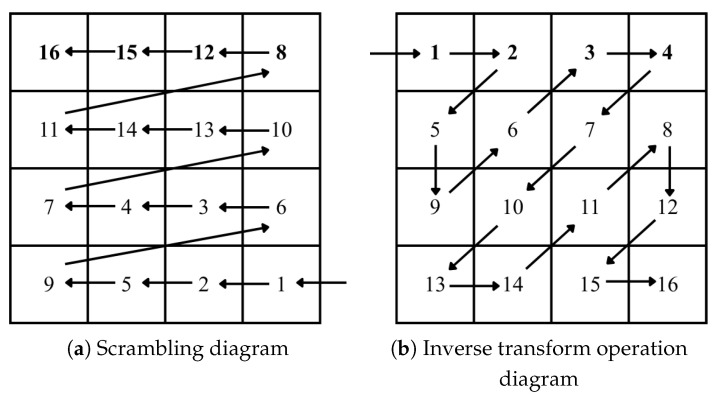
Zigzag scrambling diagram.

Step 17: To break the correlation between the three RGB channels, scramble $I_{xor}$. Because the image has 8 bits for one pixel and three channels, it can be disassembled into 24-bit planes. In this paper, we preset 24!-10 disassembling methods, such as [14, 3, 22, 9, 17, 5, 24, 12, 7, 19, 2, 16, 8, 21, 11, 4, 23, 15, 6, 13, 20, 1, 18, 10]. The selection of rules is controlled by chaotic sequences. Using the preset exchange rules, rearrange the 24-bit planes in random order. Then, within each bit plane, first use the Zigzag method to extract the bit plane into a one-dimensional bit sequence from the top-left corner. Next, place the sequence back into the bit plane according to the rule of bottom-to-top and right-to-left. Repeat this internal scrambling process three times and then the image $I_{enc}$ encrypted after the bit plane is obtained. Encryption is complete. Figure 8 is the Zigzag scrambling and inverse transform operation diagram.

### 4.2. Decryption Process

**Figure 9:**
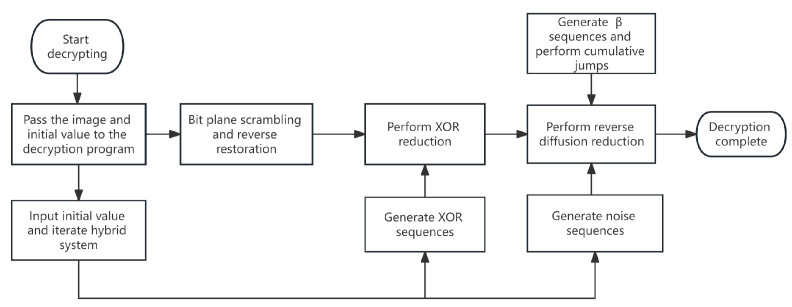
Flowchart of image decryption algorithm.

The flow chart of the decryption process is shown in Figure 9.

The decryption step is the inverse process of the encryption step. Given an M×N×3 ciphertext image $I_{enc}$, the decryption step is as follows:

Step 1: Input the ciphertext image $I_{enc}$ and the corresponding key keys.

Step 2: Follow the inverse method of bit-plane scrambling to recover to before bit-plane scrambling.

Step 3: For XOR reduction, use the key sequence keys as the initial values of the chaotic system (14), select its second state variable *y* to generate the sequence, map it to the range [0,255], and convert it to the uint8 format to obtain the XOR-sequence S of M×N×3, which is the same as that at the time of encryption. The XOR-reduced image $I_{rexor}$ is obtained by the XOR operation. XOR operation equation is as follows:

(18) $$I_{rexor}=I_{enc}⊕S.$$

Step 4: For diffusion reduction, generate a sequence β identical to the encryption using a chaotic system. Obtain the α sequence from α=1−β, obtain the value of the accumulated T steps of the α-sequence ${\bar{\alpha }}_{T}$ using ${\bar{\alpha }}_{T}=\prod_{i=1}^{T}\alpha_{i}$, and obtain the value of the accumulated T steps of the α-sequence ${\bar{\alpha }}_{T}$.

Step 5: Generate the sequence using the key sequence keys as the initial values of the chaotic system (14) and select its first state variable *x*. Because the key and the key in encryption are phase diagrammed, the same pseudo-random sequences $U_{1}$ and $U_{2}$ can be obtained.

Step 6: Generate the M×N×3 noisy image ε using the Box–Muller transform formula and normalize it to the range of [0,1]. The noisy image ε is the same as that used for encryption.

Step 7: Normalize the image $I_{rexor}$ and then use the diffusion formula corresponding to the reduction formula:

(19) $$X_{rec}=\frac{I_{rexor}-\sqrt{1-{\bar{\alpha }}_{T}}\times \varepsilon }{\sqrt{{\bar{\alpha }}_{T}}}.$$

Step 8: Obtain the decrypted and restored image $X_{rec}$, convert it back to Uint8 format, and output the decrypted and restored image $X_{rec}$.

## 5. Experimental Results

### 5.1. Experimental Platforms

The PC machine configuration was as follows: Intel(R) Core(TM) i7-6700HQ CPU @ 2.60 Ghz (8 CPUs), 16 GB of RAM, and Windows 10 64-bit OS. We wrote a single threaded program in MATLAB 2014a to implement the above simulation experiment.

### 5.2. Results

**Figure 10:**
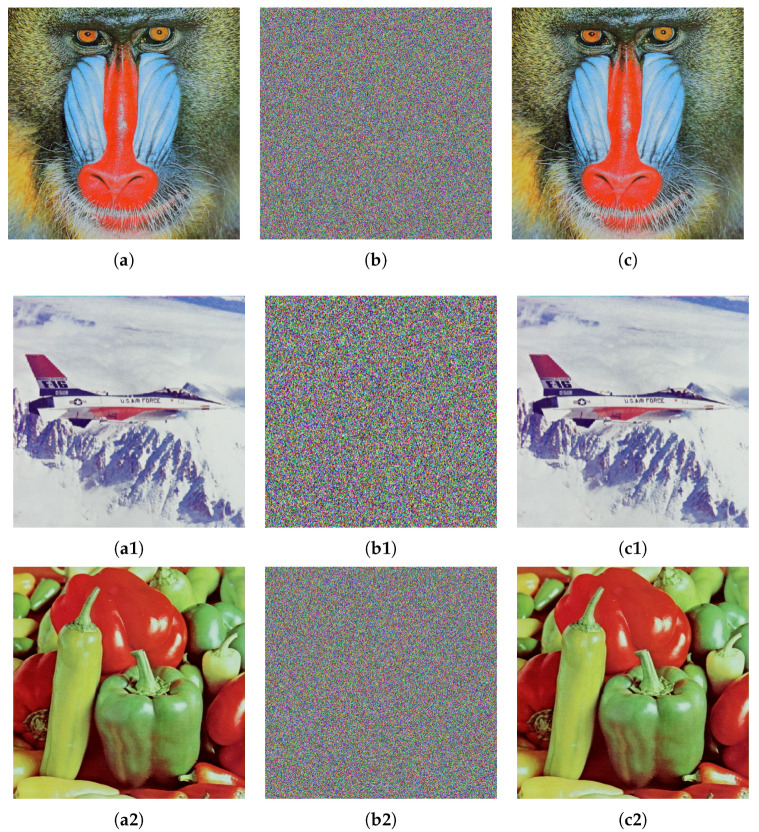
(**a**,**a1**,**a2**) plaintext images; (**b**,**b1**,**b2**) ciphertext images; and (**c**,**c1**,**c2**) decryption images.

The classic test images selected for the experiment were the color images of an Airplane (F-16), a Mandrill, and Peppers, all with a size of 512×512×3. Based on the parameters in Section 3, the plaintext image, ciphertext image, and decrypted image of the experiment are shown in Figure 10. Figure 10 shows that the algorithm achieved good encryption performance.

## 6. Security Analysis

### 6.1. Key Security Analysis

The key space is one of the most important factors for determining the strength of an image encryption algorithm. To resist an exhaustive attack, the size of the key space should be larger than $2^{128}$. In this paper, the key consists of the 256 bits of the SHA-256 function. The system parameters are a,b,c,d,e,f,g,h,i,j,k,l and the initial value of the system is $\left[key_{1},key_{2},key_{3},key_{4},key_{5},key_{6}\right]$; hence, the key space of this algorithm is $10^{270}$(calculated with a computer precision of $10^{-15}$), which is sufficient to resist an exhaustive attack. Therefore, the key security of this algorithm is sufficient. Simultaneously, the 0–1 test can effectively detect the randomness of encrypted images. The test results of the three experimental images in this study are shown in Table 2. Table 2 shows that the test values of the three images were close to 1, which indicates that the randomness of the ciphertext images was very good.

### 6.2. Chosen Plaintext Attack Analysis

**Figure 11:**
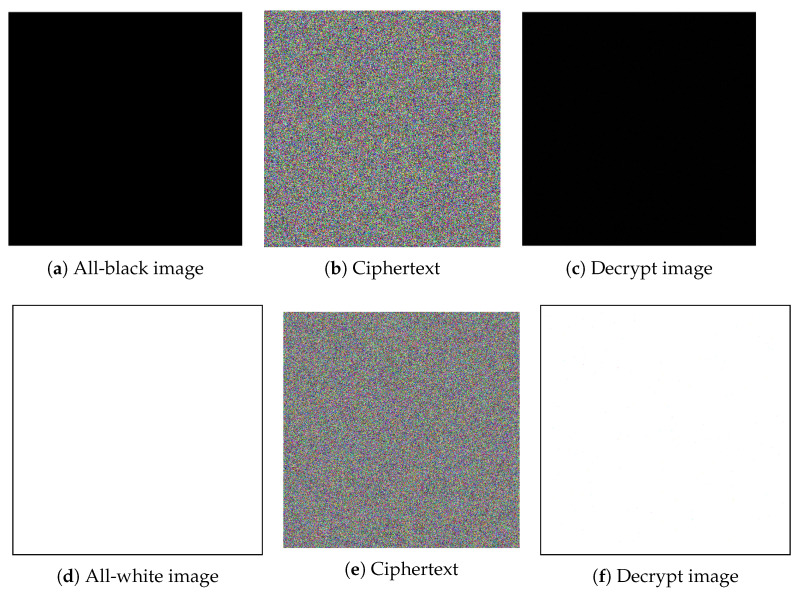
Encryption results for all-black and all-white images.

When evaluating the ability of image encryption algorithms to resist selective plaintext attacks, extreme monochrome images such as all black or all white are typically used as test plaintexts. A secure encryption algorithm should ensure that the ciphertext image of such special plaintext still exhibits pseudo-random noise characteristics, the ciphertext histogram distribution is uniform, and the information entropy is close to the theoretical maximum value of 8. In this study, we conducted encryption experiments on pure black and pure white images. The visual effect of the encrypted image is shown in Figure 11.

**Figure 12:**
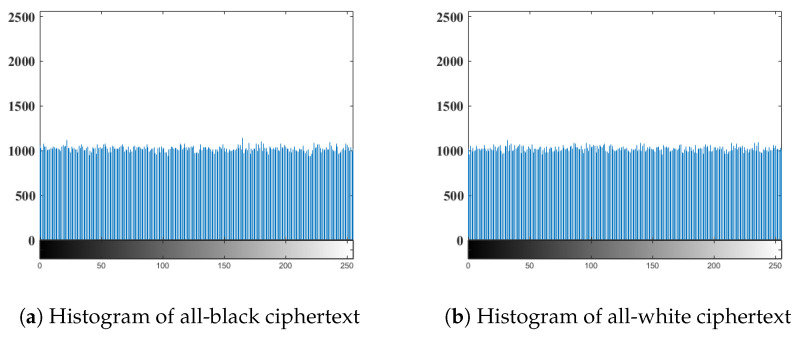
Histogram.

For further histogram analysis of the ciphertext image, taking the R channel as an example, the pixel value distribution is shown in Figure 12.

The experimental results show that the ciphertexts of pure black and pure white images exhibited a noise-like distribution visually and the histograms exhibited a high degree of uniformity. The global information entropy of both images reached 7.9993. The above qualitative and quantitative analysis fully demonstrates that the proposed algorithm effectively resisted the chosen-plaintext attack.

### 6.3. Histogram Analysis

**Figure 13:**
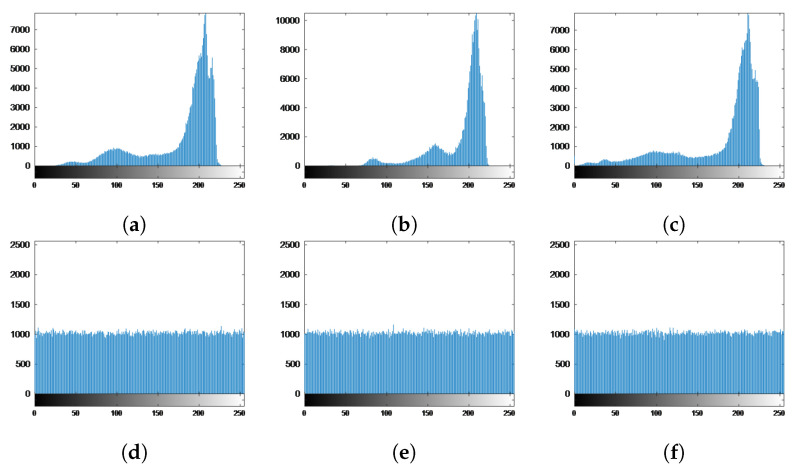
Histogram: (**a**–**c**) histograms of plaintext images; and (**d**–**f**) histograms of ciphertext images.

The histogram reflects the distribution of pixels in the region from 0 to 255 very well. When the image pixels are uniform, it can resist statistical attacks very well. The flatter the histogram appears in the region from 0 to 255, the more uniform it is. Figure 13 shows the histogram of the plaintext image and ciphertext image of the Airplane (F-16) color image. The plaintext image pixels are concentrated regularly, but after encryption, the ciphertext image pixels are evenly distributed. Hence, the encryption algorithm increases the resistance to statistical attacks.

We used a chi-square goodness-of-fit test to quantitatively analyze the uniformity of the ciphertext histogram for each channel of the three test images: Mandrill, Airplane (F-16), and Peppers. The null hypothesis of the test is that the ciphertext pixel values follow a uniform distribution, with the expected frequency set to *E*, which denotes the value of the total number of pixels divided by 256. The chi-square statistic is calculated using the formula $\chi^{2}=\sum_{i=0}^{255}\frac{O_{i}-E^{2}}{E}$, with 255 degrees of freedom. At a significance level of α=0.05, when the statistic is less than the critical value (i.e., p>0.05), the null hypothesis is accepted. As shown in Table 3, the test results for the three images across all channels satisfy p>0.05, which indicates that their ciphertext histograms all follow a uniform distribution, thereby verifying that the algorithm possesses strong resistance against statistical attacks.

### 6.4. Information Entropy Analysis

Information entropy can be used to measure uncertainty. The higher the information entropy, the greater the uncertainty of image information and the lower the amount of visual information. The formula for information entropy is

(20) $$H=-\sum_{i=1}^{L}p\left(x_{i}\right)\log_{2}p\left(x_{i}\right),$$

where $p(x_{i})$ is the probability that the value of the pixel $x_{i}$ occurs in the range 0–255. For a color image, when the gray level of the pixel’s R, G, B channel is L=256, the maximum value of information entropy is 8. The closer the information entropy of the encrypted image is to 8, the more random the encryption algorithm. A comparison of information entropy analysis is shown in Table 4.

Global information entropy may obscure the local regularities of an image. If the entropy value of a local region is low, this indicates the presence of residual low-frequency structures, which suggests insufficient encryption. To address this, in this paper, we use a sliding window to compute the local information entropy of pixel neighborhoods, thereby compensating for the limitations of global entropy detection. As shown in Table 5, the local entropy test results for all images are satisfactory, with no residual low-frequency structures, which demonstrates that the encryption algorithm is sufficiently thorough and offers high security.

### 6.5. Correlation Analysis Between Neighboring Pixel Points

To compare the adjacent pixel correlation of plaintext and ciphertext images, 5000 pairs of pixel points are randomly selected from the plaintext image and ciphertext image, and the adjacent pixel grayscale values of the horizontal, vertical, and diagonal directions of the image are analyzed. The analysis is performed using the following formula:

(21a) $$\begin{array}{c} E\left(u\right)=\frac{1}{N}\sum_{i=1}^{N}u_{i}^{2}; \end{array}$$

(21b) $$\begin{array}{c} D\left(u\right)=\frac{1}{N}\sum_{i=1}^{N}{\left(u_{i}-E\left(u\right)\right)}^{2}; \end{array}$$

(21c) $$\begin{array}{c} \mathrm{Cov}\left(u,v\right)=\frac{1}{N}\sum_{i=1}^{N}\left(u_{i}-E\left(u\right)\right)\left(v_{i}-E\left(v\right)\right); \end{array}$$

(21d) $$\begin{array}{c} r_{uv}=\frac{\mathrm{Cov}(u,v)}{\sqrt{D(u)D(v)}}. \end{array}$$

**Figure 14:**
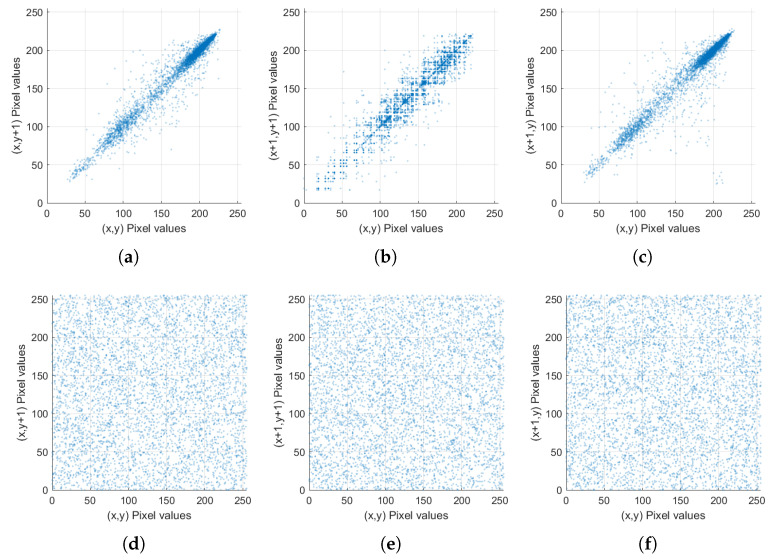
Correlation analysis diagram of the Airplane (F-16) image’s R channel in three directions before and after encryption: (**a**,**d**) diagrams of the horizontal direction; (**b**,**e**) diagrams of the vertical direction; and (**c**,**f**) diagrams of the diagonal direction.

**Figure 15:**
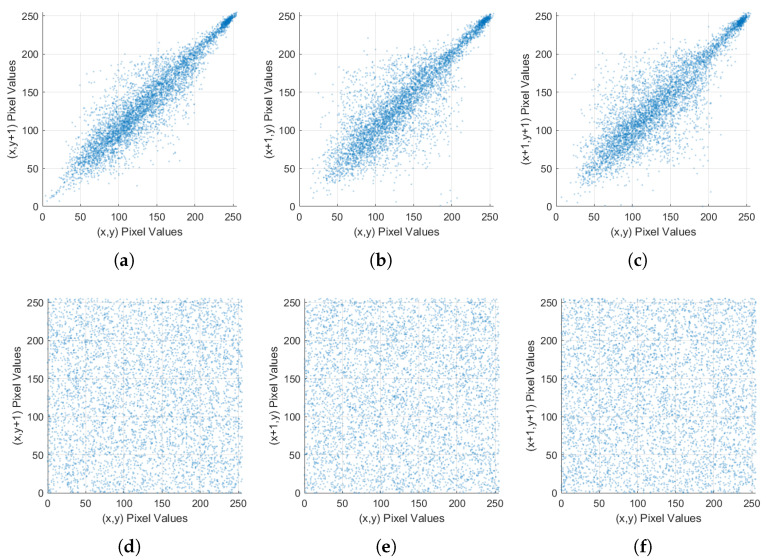
Correlation analysis diagram of the Mandrill image’s R channel in three directions before and after encryption: (**a**,**d**) diagrams of the horizontal direction; (**b**,**e**) diagrams of the vertical direction; and (**c**,**f**) diagrams of the diagonal direction.

**Figure 16:**
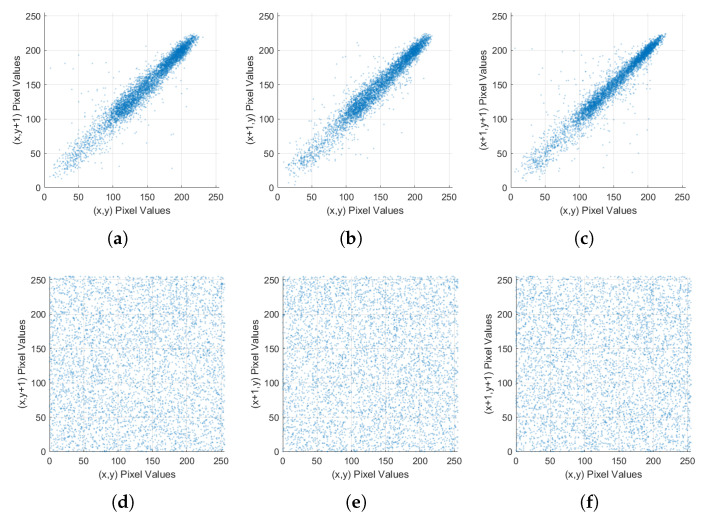
Correlation analysis diagram of the Peppers image’s R channel in three directions before and after encryption: (**a**,**d**) diagrams of the horizontal direction; (**b**,**e**) diagrams of the vertical direction; and (**c**,**f**) diagrams of the diagonal direction.

Figure 14, Figure 15 and Figure 16 show the correlation analysis of yhe Airplane (F-16), Maddrill and Peppers images before and after R-channel encryption in three directions, respectively. The figures show that the adjacent pixel gray values in the horizontal, vertical, and diagonal directions of the plaintext image exhibit obvious linear correlation, and the encrypted image does not show any obvious strong correlation of the adjacent pixel gray values in all directions.

Table 6 demonstrates the correlation coefficients of different images in all directions. Table 6 shows that the correlation coefficients of plaintext images are close to 1 in all directions and the correlation coefficients of the encrypted ciphertext images are lower than 0.002 in all directions, which indicates that the proposed encryption method achieved very good encryption performance.

### 6.6. Key Sensitivity Analysis

**Figure 17:**
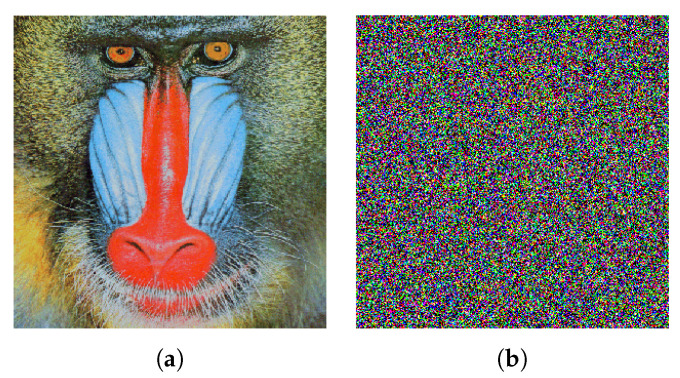
(**a**) Correct decoded image and (**b**) image decoded by slightly changed keys.

A small change in the key leads to a great difference in the ciphertext, which shows the sensitivity of the key. Take the color image of the Mandrill as an example: the key obtained by SHA-256, $key_{0}$, and $key_{1}=key_{0}+\left[0.000000000000001,0,0,0,0,0\right]$. For encryption with $key_{0}$ and decryption with $key_{0}$ and $key_{1}$, respectively, the comparison diagram is shown in Figure 17. The figure shows that there is only a small change between $key_{0}$ and $key_{1}$, but the encrypted picture can only be decrypted correctly using $key_{0}$ and cannot be decrypted correctly using $key_{1}$.

The difference between two maps can also be measured in terms of the pixel change rate (NPCR) and normalized average change intensity (UACI). The equations for NPCR and UACI are (22b) and (23), respectively:

(22a) $$D\left(i,j\right)=\left\{\begin{array}{cc} 0, & C_{1}\left(i,j\right)\neq C_{2}\left(i,j\right),\\ 1, & C_{1}\left(i,j\right)=C_{2}\left(i,j\right); \end{array}\right.$$

(22b) $$\mathrm{NPCR}=\frac{1}{M\times N}\sum_{i,j}^{M,N}D\left(i,j\right)\times 100\%;$$

(23) $$\mathrm{UACI}=\frac{\sum_{i,j}^{M,N}\left|C_{1}\left(i,j\right)-C_{2}\left(i,j\right)\right|}{M\times N\times 255}\times 100\%.$$

where *M* and *N* are the sizes of the images, and $C_{1}\left(i,j\right)$ and $C_{2}\left(i,j\right)$ are the pixel values of the two images at the position of (i,j), respectively. The greater the value of the NPCR and UACI, the larger the difference between the two images. The theoretical values of NPCR and UACI are 99.6094% and 33.4635%, respectively; that is, the closer to the ideal value, the greater the difference between the two images.

In this study, we encrypted the classic test image, 512×512×3 pixels, color Mandrill, using $key_{0}$ and $key_{1}$ to obtain the NPCR and UACI values of the two cipher images, respectively. We made minor modifications to the six initial values, with a modification value of 0.000000000000001. The test results are shown in Table 7.

Table 7 shows that the values of NPCR and UACI for different test images are close to the ideal values; hence, the proposed key system has good key sensitivity.

### 6.7. Differential Attack Analysis

In a differential attack, the value attacker compares the difference between the encrypted ciphertext of the original image and the encrypted ciphertext of the changed image by slightly changing the plaintext, and then determines the relationship between the corresponding plaintext and ciphertext images. NPCR and UACI metrics can also be used to test the ability of image encryption schemes against differential attacks. We conducted differential attack analysis on the Airplane (F-16), Mandrill, and Peppers images by modifying one pixel, one row, one column, an 8×8 block, flipping the highest bit of one pixel, flipping the middle bit of one pixel, flipping the lowest bit of one pixel, and swapping the R and B channels. The test results are shown in Table 8. Table 8 shows that the NPCR of the encrypted images are all close to 99.5597% and the UACI are close to 33.4079%. Table 9 is its comparison table.

### 6.8. Anti-Noise Attack

**Figure 18:**
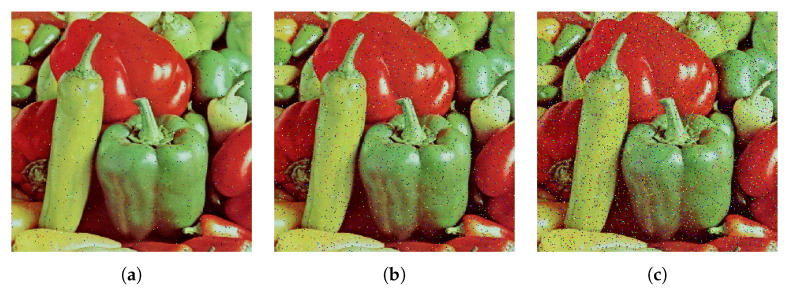
Anti-noise attack: (**a**,**b**,**c**) decrypted images with noise strengths 0.01, 0.03, and 0.06, respectively.

**Figure 19:**
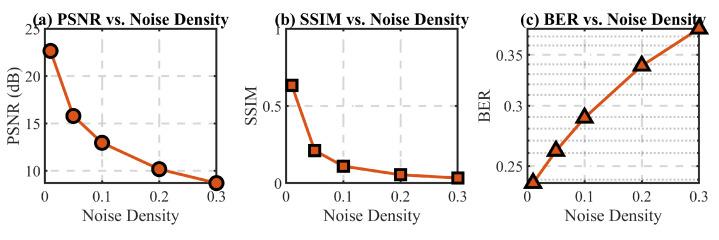
Bit-error rate as a function of noise density.

In the process of encrypted image transmission, the image can be damaged by noise interference. Additionally, the attacker can use the noise attack to destroy the integrity of the encrypted image and prevent the receiver from decrypting it successfully. Taking the plaintext image Peppers as an example, we simulated a noise attack on its ciphertext image with different degrees and obtained a slightly different decrypted image after decryption. The test results are shown in Figure 18. After the ciphertext image is added with the salt-and-pepper noise with the noise strength of 0.01, 0.03 and 0.06, the decrypted images are shown in Figure 18 from the left to the right, in order. PSNR, SSIM and bit-error rate analysis are shown in Table 10 and Figure 19.

### 6.9. Anti-Clipping Attack

**Figure 20:**
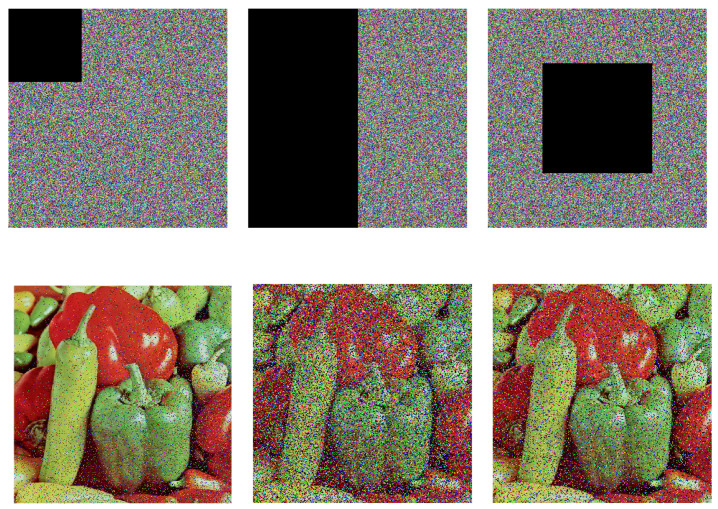
Anti-cropping attack.

**Figure 21:**
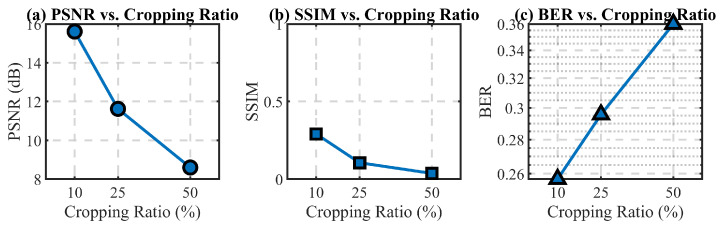
Bit-error rate as a function of cropped area.

In the process of ciphertext image transmission, part of the image information may be cropped by the attacker, thereby resulting in information integrity being affected or even a scenario in which the receiver cannot quickly decrypt the correct image information. A secure encryption algorithm can resist the cropping attack effectively. The test results for the ciphertext image of the plaintext image Peppers under a data cropping attack are shown in Figure 20. The results clearly show that the proposed algorithm decrypted the plaintext image successfully after different degrees of cropping attacks and retained the original text information well, with high security and robustness. Figure 20 shows three different cropping attacks: corner data cropping, $\frac{1}{2}$-data cropping, and center data cropping. PSNR, SSIM and cropped area analysis are shown in Figure 21 and Table 11.

### 6.10. Computational Complexity and Time Analysis

To evaluate the computational efficiency of the algorithm, we conducted single-threaded encryption and decryption experiments on images of three sizes (128×128×3, 256×256×3, and 512×512×3) in the environment of I7 6700HQ CPU, Windows 10, and MATLAB. As shown in Table 12, when the pixel count increased by a factor of 4, the average encryption time rose from 0.1663 s to 0.6583 s (approximately 3.95 times) and from 0.6583 s to 2.5771 s (approximately 3.91 times), whereas the decryption time growth rates were 3.78 and 3.93, respectively, with trends largely consistent with encryption. The time growth rates closely approximate the pixel growth rates, which indicates that the proposed algorithm’s time complexity is O(*N*) (where *N* is the total number of pixels), which means that each pixel undergoes only O(1) basic operations. Additionally, the encryption and decryption time consumption and growth trends exhibited high symmetry, which validates the algorithm’s structural symmetry and consistency. To summarize, the proposed algorithm has low time complexity and can meet the processing efficiency requirements in practical applications.

## 7. Conclusions

In this paper, we proposed a new hyperchaotic system and constructed a new image encryption scheme by combining the forward diffusion process of SHA-256 and DDPM. We conducted a theoretical analysis and numerical simulation experiments to characterize the basic dynamics of this chaotic system, including an attractor, Lyapunov exponential spectrum, time series diagram, phase diagram, and Poincare section. The relevant analysis in the study indicates that the system is hyperchaotic.

Using SHA-256 to link image information with key acquisition, and combining the sensitivity of chaotic systems to initial values and the unpredictability of chaotic sequences, a hyperchaotic sequence that follows the Gaussian noise distribution during the diffusion stage can be generated, which can increase the randomness of ciphertext images. In the encryption process, to further increase the randomness of the ciphertext image, we combined the DDPM forward diffusion method with the skip mechanism of the Markov chain to diffuse the hyperchaotic sequence following the Gaussian noise distribution into the plaintext image, thereby achieving the goal of changing pixel values. Then, we used the pseudo-random sequence generated by the chaotic system for the XOR encryption operation to further encrypt the image. Finally, we independently scrambled the 24-bit planes using the bit plane scrambling method. The simulation experiment results and security analysis show that this method achieved good encryption performance.

## Figures and Tables

**Table 1:** 0–1 Test Values.

Hyper Chaotic Sequences	*x*	*y*	*z*	*u*	*v*	*w*
0−1 test values	0.9976	0.9984	0.9976	0.9981	0.9973	0.9976

**Table 2:** 0–1 Test Values.

Image	Mandrill	Airplane (F-16)	Peppers
0–1 test values	0.9976	0.9978	0.9980

**Table 3:** Chi-square goodness-of-fit test for ciphertext images under the assumption of a uniform distribution.

Ciphertext Image	Channel	Test Statistic ($\;\chi^{2}$)	*p*-Value	Critical Value (α=0.05)	Result
Mandrill	R	220.7148	0.940884	293.2478	Uniform
	G	238.6094	0.761849	293.2478	Uniform
	B	268.5313	0.268192	293.2478	Uniform
Airplane (F-16)	R	262.7520	0.355953	293.2478	Uniform
	G	245.8594	0.648056	293.2478	Uniform
	B	262.8340	0.354628	293.2478	Uniform
Peppers	R	233.4863	0.829222	293.2478	Uniform
	G	251.6836	0.546941	293.2478	Uniform
	B	260.8711	0.386873	293.2478	Uniform

**Table 4:** Information entropy comparison.

Algorithm	Airplane (F-16) Image			Mandrill Image			Peppers Image		
	**R**	**G**	**B**	**R**	**G**	**B**	**R**	**G**	**B**
Original figure	7.2531	7.5940	6.9684	6.9294	6.3175	7.2895	6.3319	6.4072	6.1137
This text	7.9992	7.9993	7.9993	7.9992	7.9993	7.9992	7.9994	7.9992	7.9994
Ref. [19]	7.9992	7.9992	7.9992	7.9992	7.9992	7.9992	7.9993	7.9992	7.9990
Ref. [20]	–	–	–	7.9993	7.9993	7.9993	7.9992	7.9994	7.9994
Ref. [21]	7.9993	7.9993	7.9993	7.9993	7.9993	7.9993	7.9993	7.9993	7.9993
Ref. [22]	–	–	–	7.9982	7.9988	7.9985	7.9983	7.9983	7.9971

**Table 5:** Local information entropy of multiple window sizes.

Channel	Image	8×8 Window		16×16 Window		32×32 Window	
		**Mean**	**Std**	**Mean**	**Std**	**Mean**	**Std**
Mandrill	R	5.7650	0.0770	7.1740	0.0525	7.8088	0.0176
	G	5.7667	0.0764	7.1770	0.0524	7.8095	0.0176
	B	5.7635	0.0773	7.1726	0.0529	7.8081	0.0171
Airplane (F-16)	R	5.7641	0.0767	7.1724	0.0525	7.8071	0.0176
	G	5.7656	0.0762	7.1736	0.0528	7.8076	0.0174
	B	5.7644	0.0770	7.1736	0.0532	7.8078	0.0180
Peppers	R	5.7674	0.0764	7.1769	0.0520	7.8108	0.0169
	G	5.7647	0.0766	7.1744	0.0531	7.8089	0.0171
	B	5.7650	0.0769	7.1747	0.0524	7.8097	0.0170

**Table 6:** Plaintext image and ciphertext image correlation coefficient test result table.

Image	Channel	Plaintext Image			Ciphertext Image		
		**Horizontal**	**Vertically**	**Diagonal**	**Horizontal**	**Vertically**	**Diagonal**
Airplane (F-16)	R	0.9746	0.9469	0.9327	−0.0142	0.0055	0.0080
	G	0.9543	0.9677	0.9334	−0.0022	0.0090	0.0233
	B	0.9633	0.9303	0.9183	−0.0224	0.0105	−0.0073
Mandrill	R	0.9062	0.8572	0.8469	−0.0186	0.0230	0.0097
	G	0.8665	0.7790	0.7442	−0.0058	−0.0202	0.0290
	B	0.8985	0.8695	0.8209	0.0215	0.0012	−0.0007
Peppers	R	0.9596	0.9648	0.9564	−0.0145	0.0165	0.0125
	G	0.9836	0.9838	0.9760	−0.0010	−0.0092	−0.0174
	B	0.9588	0.9619	0.9471	−0.0084	−0.0109	−0.0082

**Table 7:** NPCR and UACI values of encrypted images with slight key changes.

Image	NPCR	UACI
Mandrill (1)	99.6102%	33.4741%
Mandrill (2)	99.6089%	33.4694%
Mandrill (3)	99.6105%	33.4801%
Mandrill (4)	99.6108%	33.4699%
Mandrill (5)	99.6111%	33.4723%
Mandrill (6)	99.6097%	33.4709%
Peppers	99.5938%	33.4473%
Airplane (F-16)	99.6088%	33.4575%

**Table 8:** Analysis of multi-mode differential attacks.

Image	Airplane (F-16)		Mandrill		Peppers	
	**NPCR**	**UACI**	**NPCR**	**UACI**	**NPCR**	**UACI**
Single Pixel	99.6033%	33.4856%	99.6118%	33.4854%	99.6154%	33.4725%
Single Row	99.6127%	33.4783%	99.5995%	33.4576%	99.6096%	33.4988%
Single Col	99.6049%	33.4357%	99.6296%	33.4857%	99.6114%	33.4784%
Block 8 × 8	99.6082%	33.4404%	99.6114%	33.4800%	99.6104%	33.4781%
MSB Flip	99.6154%	33.4689%	99.6096%	33.4251%	99.6115%	33.4876%
Middle Bit Flip	99.6136%	33.4835%	99.6104%	33.4593%	99.6051%	33.4292%
LSB Flip	99.6155%	33.4519%	99.6115%	33.4079%	99.6146%	33.5079%
Channel Swap	99.6146%	33.4505%	99.6051%	33.4150%	99.6096%	33.4561%

**Table 9:** Analysis of an anti-differential attack.

Image	Airplane (F-16)		Mandrill		Peppers	
	**NPCR**	**UACI**	**NPCR**	**UACI**	**NPCR**	**UACI**
Theoretical value of indicator	99.6094%	33.4635%	99.6094%	33.4635%	99.6094%	33.4635%
This text	99.6050%	33.4628%	99.6125%	33.4887%	99.6035%	33.4554%
Ref. [23]	99.6523%	33.4557%	–	–	33.6320%	33.4518%
Ref. [24]	99.6350%	33.5158%	99.6294%	33.4615%	99.6396%	33.4780%
Ref. [25]	99.6573%	33.4656%	99.6603%	33.4659%	99.6284%	33.4638%
Ref. [26]	99.6103%	33.4754%	99.6115%	33.4526%	99.6063%	33.4729%

**Table 10:** Noise density analysis.

Noise Density	PSNR (dB)	SSIM	BER
0.01	22.6558	0.6332	0.237479
0.05	15.7859	0.2110	0.261704
0.10	12.9533	0.1093	0.289281
0.20	10.1694	0.0536	0.338536
0.30	8.7041	0.0329	0.378671

**Table 11:** Cropped area analysis.

Cropping Ratio	PSNR (dB)	SSIM	BER
0.10	15.6104	0.2910	0.256994
0.25	11.6227	0.1055	0.295690
0.50	8.5979	0.0368	0.360005

**Table 12:** Encrypted and decrypted time analysis.

Image	Image Size	Encryption	Decryption
Airplane (F-16)	128×128×3	0.1667 s	0.1688 s
	256×256×3	0.6396 s	0.6263 s
	512×512×3	2.7217 s	2.7579 s
Mandrill	128×128×3	0.1666 s	0.1641 s
	256×256×3	0.6821 s	0.6531 s
	512×512×3	2.4975 s	2.3731 s
Mandrill Ref. [22]	512×512×3	3.7385 s	–
Pepper s	128×128×3	0.1657 s	0.1681 s
	256×256×3	0.6532 s	0.6181 s
	512×512×3	2.5123 s	2.4916 s
Peppers Ref. [22]	512×512×3	3.6686 s	–

## Data Availability

Data will be made available on request.
